# Inferring Allele Frequency Trajectories from Ancient DNA Indicates That Selection on a Chicken Gene Coincided with Changes in Medieval Husbandry Practices

**DOI:** 10.1093/molbev/msx142

**Published:** 2017-05-02

**Authors:** Liisa Loog, Mark G. Thomas, Ross Barnett, Richard Allen, Naomi Sykes, Ptolemaios D. Paxinos, Ophélie Lebrasseur, Keith Dobney, Joris Peters, Andrea Manica, Greger Larson, Anders Eriksson

**Affiliations:** 1 The Palaeogenomics and Bio-Archaeology Research Network, Research Laboratory for Archaeology and History of Art, University of Oxford, Oxford, United Kingdom; 2 Department of Zoology, University of Cambridge, Cambridge, United Kingdom; 3 Research Department of Genetics, Evolution and Environment, University College London, London, United Kingdom; 4 Department of Archaeology, University of Nottingham, Nottingham, United Kingdom; 5 Department of Veterinary Sciences, Institute of Palaeoanatomy, Domestication Research and the History of Veterinary Medicine, LMU Munich, Munich, Germany; 6 Department of Archaeology, School of Geosciences, University of Aberdeen, St. Mary’s, United Kingdom; 7 Department of Archaeology, Classics and Egyptology, University of Liverpool, Liverpool, United Kingdom; 8 Department of Archaeology, Simon Fraser University, Burnaby, Canada; 9 SNSB, Bavarian State Collection of Anthropology and Palaeoanatomy, Munich, Germany; 10 Department of Medical & Molecular Genetics, King's College London, Guys Hospital, London, United Kingdom

**Keywords:** ancient DNA, selection, Bayesian analyses, TSHR, BCDO2, domestication

## Abstract

Ancient DNA provides an opportunity to infer the drivers of natural selection by linking allele frequency changes to temporal shifts in environment or cultural practices. However, analyses have often been hampered by uneven sampling and uncertainties in sample dating, as well as being confounded by demographic processes. Here, we present a Bayesian statistical framework for quantifying the timing and strength of selection using ancient DNA that explicitly addresses these challenges. We applied this method to time series data for two loci: TSHR and BCDO2, both hypothesised to have undergone strong and recent selection in domestic chickens. The derived variant in TSHR, associated with reduced aggression to conspecifics and faster onset of egg laying, shows strong selection beginning around 1,100 years ago, coincident with archaeological evidence for intensified chicken production and documented changes in egg and chicken consumption. To our knowledge, this is the first example of preindustrial domesticate trait selection in response to a historically attested cultural shift in food preference. For BCDO2, we find support for selection, but demonstrate that the recent rise in allele frequency could also have been driven by gene flow from imported Asian chickens during more recent breed formations. Our findings highlight that traits found ubiquitously in modern domestic species may not necessarily have originated during the early stages of domestication. In addition, our results demonstrate the importance of precise estimation of allele frequency trajectories through time for understanding the drivers of selection.

## Introduction

A comprehensive understanding of the evolutionary responses to changing selection pressures can be achieved by reconstructing allele frequency trajectories through time and linking them to changes in the ecological context in which an organism has evolved. This association has typically only been possible in longitudinal genomic studies of laboratory organisms with relatively short generation times, whose environments can be easily and rapidly manipulated (Kawecki et al. 2012). Linking episodes of selection to their drivers in natural systems is more challenging. The most common approach has been to study large sets of modern genetic data from closely related species or subpopulations using various statistical approaches (e.g., genome wide scans for selection) (Storz 2005). However, these approaches typically lack the power to reliably estimate the timing and strength of selection, especially over short evolutionary time scales. Consequently, it is challenging to associate past episodes of selection with specific drivers, especially when those drivers are not present in contemporary environments.

Ancient DNA data can provide direct information on changes in allele frequencies through time, and as such allows us to directly link past selection to contemporaneous ecological factors. However, ancient DNA sample sizes are typically small, and samples tend to be sparsely and unevenly distributed in space and time. Several methods exist for studying selection using ancient DNA (reviewed in Malaspinas 2016), but typically they lack the ability to model the confounding effects of gene flow—a process that could lead to overestimation of selection coefficients (Mathieson et al. 2015). To overcome these challenges, we developed a Bayesian statistical framework that permits formal quantification of selection parameters using ancient allele frequency data, while explicitly taking into account both uncertainty in sample ages and gene flow from external sources, and apply this framework to genotype data from domestic chicken.

The domestication of chickens (*Gallus gallus domesticus*) from wild junglefowl took place over a relatively short evolutionary timeframe (Rubin et al. 2010). In addition, the genetic architectures underlying many of the phenotypic traits that distinguish domestic populations are well understood (Eriksson et al. 2008; Rubin et al. 2010). As such, chickens present an ideal opportunity to correlate the timing of allele frequencies shifts with changes in human imposed selective pressures over the past several thousand years. To do so, we focused on two loci: thyroid-stimulating hormone receptor (*TSHR*) and β-carotene dioxygenase 2 (*BCDO2*), both previously identified as targets of selection in domesticated chickens (Rubin et al. 2010).

*TSHR* plays a role in growth, metabolic regulation and photoperiod control of reproduction in mammals and birds by stimulating the synthesis and release of thyroid hormones (Yoshimura et al. 2003; Hanon et al. 2008; Nakao et al. 2008; Follett 2015; Nishiwaki-Ohkawa and Yoshimura 2016). Modern chickens carry a derived recessive *TSHR* allele that is thought to cause loss of strict seasonal reproduction (Rubin et al. 2010)—a commonly observed difference between domesticated animals and their wild relatives (Belyaev 1979)—and has been shown to associate with faster onset of egg laying at sexual maturity (Karlsson et al. 2016). This allele has also been directly associated with reduced aggressive behaviors toward conspecifics and decreased fear of humans (Karlsson et al. 2015). It has, therefore, been suggested that these traits could have been selected to increase egg yield and facilitate larger groups or higher bird densities in a domestic setting (Karlsson et al. 2015, 2016).

The *BCDO2* gene is expressed in the skin where it encodes an enzyme that cleaves colorful carotenoids into colorless apocarotenoids, and polymorphisms in the *BCDO2* gene have well-known effects on skin pigmentation in birds (Eriksson et al. 2008). Domestic chickens are known to possess both a dominant, ancestral *BCDO2* allele that results in white or grey skin, and a recessive derived allele that is associated with yellow skin (Eriksson et al. 2008)—the latter likely acquired by domestic chickens through admixture with grey jungle fowl (*Gallus sonneratii*) (Eriksson et al. 2008). The biological mechanism for selection at the BCD02 locus is less clear than for the TSHR locus, but it has been previously hypothesised that the yellow legs phenotype could have been used as a proxy for good nutritional status in chickens, or selected for purely cosmetic reasons (Eriksson et al. 2008).

Genome-wide comparison between domesticated chickens and their wild relatives provides a powerful method for identifying loci under selection since chickens were domesticated around 6,000 years ago (Peters et al. 2016). Given the ubiquity of the derived TSHR allele in modern domesticated chicken populations (Rubin et al. 2010), and its association with several hallmark domestication traits (Karlsson et al. 2015), it has been proposed that the derived TSHR allele was selected during the initial stages of the domestication process (Rubin et al. 2010; Karlsson et al. 2015). However, due to the low mutation and recombination rates in nuclear DNA, these studies lack the power to accurately estimate the timing and the strength of selection.

**Fig. 1.**
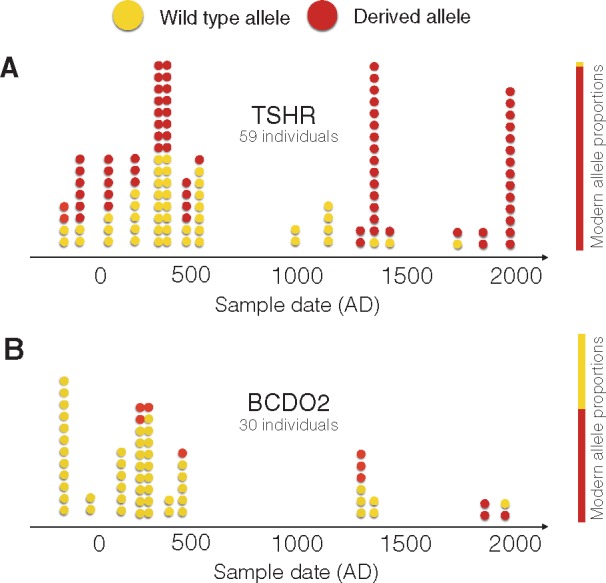
Observed allele counts through time for the TSHR locus (*a*) and the BCDO2 locus (*b*). Wild type alleles in ancient samples are represented by yellow dots and derived alleles by red dots. Modern allele proportions are shown as solid bars to the right of each panel.

Flink et al. (2014) typed the TSHR and BCDO2 loci in archaeological chicken samples from Europe, spanning the last 2,200 years, to further examine the hypothesis of selection during early domestication. They observed significantly lower derived allele frequencies in their ancient samples relative to the modern populations, which led them to reject the early selection hypotheses for both loci in favor of more recent selection. Given the sparseness and uneven temporal distribution of their data, they did not attempt to quantify the timing or strength of selection. A further complication to the analysis of selection comes from recent gene flow in domesticated chickens. During modern European chicken breed formation (beginning 100–150 years ago), Asian chickens were imported and bred with local European stock to improve existing birds and create novel breeds (Dana et al. 2011; Flink et al. 2014; Lyimo et al. 2015). In this study, we applied our statistical framework to the ancient DNA data reported by Flink et al. (2014) and 16 additional samples (fig. 1 and Materials and Methods), and used forward simulations to test if genetic drift and gene flow, without invoking selection, could explain the observed allele frequency changes.

## New Approaches

### A Bayesian Framework to Infer Past Episodic Selection

We represented the European domesticated chickens as a randomly mating population (an assumption we test below) in which an allele is under selection, and that receives gene flow from an external source. To simplify the model, we assumed that the population size was large enough to ignore genetic drift on these time scales. This allowed us to treat allele frequency trajectories as deterministic, an assumption that we also tested. Combining the standard model for selection on a recessive trait with a term that corresponds to gene flow from an external population, we obtained a differential equation for the allele frequency trajectory of the selected allele,

(Eq. 1) $$\frac{df}{dt}=s\left(t\right)f^{2}\left(1-f\right)+m(t)(f_{\text{external}}-f),$$

where *f* is the frequency of the hypothesized selected allele, *s* is the selection coefficient, *m* is the rate of gene flow and *f*_external_ is the frequency of hypothesized selected allele in the external source population. The initial value for *f*(t) is the ancestral frequency (*f*_ancestral_). The model has three additional parameters defining the starting time of selection (*t*_selection_) and the beginning (*g*_start_) and end (*g*_end_) of gene flow from the external source. The selection coefficient *s* is constant after the starting time and is zero before. Similarly, *m* is constant in the gene flow interval and is zero at other times.

Using only the genotype information to estimate all seven parameters of the model would be challenging. However, because chickens are a relatively well-studied species, we were able to specify the values of four parameters by using historic information and mitochondrial DNA data, thus greatly reducing model search space. Gene flow from Asia is historically well-documented (Dana et al. 2011; Flink et al. 2014; Lyimo et al. 2015), beginning around 250 years ago (*g*_start_ = 250) and continuing until the present day (*g*_end_ = 0). The rate of gene flow *m* was estimated using modern and ancient mitochondrial haplotype frequencies, taking advantage of the fact that all ancient European chickens (i.e., prior to the gene flow from Asia) have the same mitochondrial haplotype (see Materials and Methods). The frequency of the derived allele in the Asian population at the time of gene flow is currently unknown. To minimize the possibility of overestimating the role of selection in increasing the allele frequency over the effect of gene flow from Asia, we made a conservative assumption that *f*_external_ = 0.99 (i.e., the selected allele is nearly fixed in Asian chickens).

The likelihood of the data is a function of the frequency curve of the selected allele in the population and is calculated as the product of the probabilities of all observed alleles:

(Eq. 2) $$L=\prod_{i}f{\left(t_{i}\right)}^{x_{i}}{\left(1-f\left(t_{i}\right)\right)}^{1-x_{i}}.$$

Here *L* is the likelihood of the data, *f*(*t*) is the allele frequency at the time *t*, *t_i_* is the age of the sample *i*, and *x*_i_ = 1 in case of observing the derived allele in sample *i*, and *x*_i_ = 0 when observing the wild type.

Equation (2) assumes that sample ages are precisely known. However, archaeological samples often come with some age uncertainty. To account for this, we replace the likelihood of each genotype (each factor in eq. 2) with its posterior likelihood given the distribution of sample age from archaeological dating (see Materials and Methods).

## Results

### Timing and Strength of Selection, and Past Allele Frequency Trajectories for *TSHR* and *BCDO2*

The remaining parameters of the model—the starting time of selection (*t*_selection_), the selection coefficient (s) and the ancestral frequency of the selected allele (*f*_ancestral_)—were inferred by making a full parameter sweep where we calculated the deterministic allele frequency trajectory for each parameter combination. We then calculated the posterior probability density distribution for each parameter by numerically integrating the likelihood of the data over the remaining two parameters (assuming a uniform prior for starting time of selection, ancestral frequency, and the logarithm of the selection strength). In addition, to visualize the inferred allele frequency trajectories, we calculated the posterior distribution of allele frequency through time by weighting the allele frequency trajectory of each parameter combination (sampled from their prior distributions) by the likelihood of the data given the parameters.

**Fig. 2.**
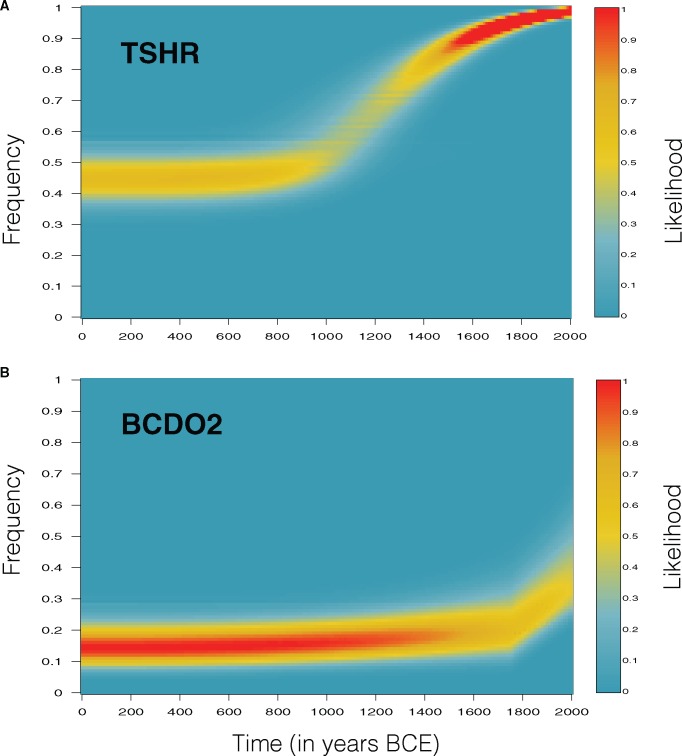
Posterior distribution of the derived allele frequency as a function of time for the TSHR locus (*a*) and the BCDO2 locus (*b*). The likelihoods are color coded (see color bar) and are shown relative to the maximum likelihood in the plot.

For the TSHR locus, we estimated a selection coefficient of 0.0049 (95% CI: 0.0030–0.0069, supplementary fig. S1, Supplementary Material online), which would have generated a rapid increase in allele frequency (fig. 2*a*) starting 920 AD (95% CI: 290–1210 AD, supplementary fig. S1, Supplementary Material online). We also inferred that the frequency of the TSHR derived allele prior to selection in the ancestral chicken population was 0.44 (95% CI: 0.34–0.54, supplementary fig. S1, Supplementary Material online), similar to that estimated in a red jungle fowl captive zoo population (frequency 0.35, 95% CI:0.22–0.50 (Rubin et al. 2010)).

For the BCDO2 locus, we inferred a selection coefficient of 0.0036 (95% CI: 0.0001–0.0158, supplementary fig. S2, Supplementary Material online), similar in magnitude to that estimated for the TSHR locus. Despite supporting selection, the inferred allele frequency trajectories corresponding to the best fitting parameter combinations show only a very marginal increase in the BCDO2 derived allele frequency prior to the time of gene flow (fig. 2*b*), therefore the observed rise in allele frequency can be largely attributed to recent Asian gene flow. This result is expected given the low inferred ancestral frequency of the derived allele (*f*_ancestral_=0.14, 95% CI: 0.07–0.25, supplementary fig. S2, Supplementary Material online), as the efficacy of selection for a recessive adaptive allele is highly sensitive to its initial frequency. As a consequence of minimal increase in allele frequency prior to the period of Asian gene flow, the starting time of selection is poorly resolved in this analysis (supplementary fig. S2, Supplementary Material online), and therefore, has little effect on the allele frequency trajectory.

Analyses of selection usually focus on estimating the strength of selection, and on rejecting the null model of genetic drift as an explanation of the data (Malaspinas 2016). However, selection coefficients can be difficult to interpret as the inferred values do not necessary reflect the true rate of change in allele frequencies, especially for recessive alleles—we inferred similar selection coefficients for the TSHR and BCDO2 genes, but while the former shows a rapid increase in allele frequency, the latter results in only very marginal rate of change in allele frequencies. Thus, genotype data spanning an episode of selection, combined with statistical modeling, allow precise estimation of allele frequency trajectories though time (e.g., fig. 2). This in turn enables direct examination of both the timing as well as rate of adaptation, and greatly simplifies the biological interpretation of the effect of selection during the episode.

### Testing the Confounding Effect of Genetic Drift

To test if the observed data could be explained by purely demographic factors, such as the rate of genetic drift and recent Asian gene flow, we simulated 10,000 stochastic allele frequency trajectories under this model (see Materials and Methods). We then estimated the proportion of likelihood values greater than or equal to the values from the deterministic model of selection and gene flow (for a formal significance test), as well as the average likelihood of the trajectories (for a formal likelihood ratio test). To minimize the possibility of underestimating the role of drift in increasing the allele frequency over that of selection, we took a conservative approach and used BEAST (Drummond et al. 2012) to estimate the effective population size of the European chicken population based on mtDNA sequences from modern samples (see Material and Methods). Since the male to female ratio in the ancient European chicken population is unknown, we assumed the size of the female effective population size as the effective populations size in our stochastic simulations.

For the TSHR locus, all of the stochastic trajectories from the drift model had a lower likelihood than the best fitting trajectory from the selection model. In addition, the formal likelihood ratio test estimates the selection model to be 3 × 10^19^ times more likely than the drift model. Consequently, the high frequency of the derived allele in the modern domesticated chicken populations cannot be explained by drift and gene flow alone, despite our conservative assumptions.

For the BCDO2 derived allele, the best fitting selection model explains the data only marginally better than the drift model. Out of the 10,000 simulated allele frequency trajectories, 366 have a likelihood value as high or higher than the best fitting selection model, and the selection model is nine times more likely than the drift model. Thus, while it is possible to obtain the patterns observed in the ancient DNA under the drift model, it is rare and the selection model is still supported by the data.

Finally, to test the effect of gene flow on our ability to reject selection over genetic drift, we ran the deterministic and stochastic models without migration. As expected from the timing and strength of selection acting on the TSHR locus, removing gene flow from the stochastic model made little difference to our ability to reject the null-hypothesis of no selection (*P* < 0.0001). In contrast, selection for the BCDO2 locus became highly significant (*s* = 0.0060, *P* = 0.0005) in models without migration, confirming that unaccounted gene flow can cause spurious effects in selection analyses.

### Sensitivity Analyses

As discussed in the introduction, sparseness of samples and geographic patterns could confound analysis of past selection. To assess whether geographic structure in the data could affect the analysis, we first applied a standard Hardy–Weinberg test to ancient samples predating the Medieval times, and fail to reject Hardy–Weinberg equilibrium for both TSHR (χ^2^ = 0.057, *P *=* *0.81) and BCD02 (χ^2^ = 0.20, *P *=* *0.66) (seeMaterials and Methods). This lack of structure is also consistent with the observation that European ancient chicken samples have the same mitochondrial haplotype (Flink et al. 2014). As an explicit test of spatial genetic and cultural differences across Europe, we restricted the data to locations from Northwest Europe and excluded samples from the Mediterranean region (see Materials and Methods and supplementary fig. S4, Supplementary Material online). Analysis on this subset of the data yielded parameter ranges similar to the full data set (supplementary table S9, Supplementary Material online), suggesting little or no effect of geographic structure on our estimates. The main difference between the two results is that the starting time and coefficient of selection are less well defined in the analyses with the restricted data compared to the analysis with the full data set (supplementary tables S9 and figs. S5 and S6, Supplementary Material online). For TSHR, we also observed an increased uncertainty of inferred allele frequency trajectory between 1100 and 1600 AD (supplementary fig. S7, Supplementary Material online). This is mostly due to the fact that the analysis with the reduced data set lacks most samples from this informative time period. However, the increase in allele frequency in the 10th century AD is still best supported, and we can confidently rule out a significant rise in allele frequency prior to 900 AD due to the large number of samples predating the period (supplementary fig. S7, Supplementary Material online).

To explore the effect of sparseness of ancient samples, we picked a parameter combination similar to the best supported scenario for TSHR, simulated 50 replicate data sets with sample sizes ranging from 10 to 200 diploid individuals (see Materials and Methods), and repeated the analysis for each data set. Supplementary figure S8, Supplementary Material online shows the confidence intervals for the three estimated parameters in each data set. As expected, we observe a trend of increasingly wide confidence intervals with decreasing sample size. However, the medians of the posterior distributions are scattered near the true value, indicating little or no bias in estimating the parameters even for relatively small number of sample sizes.

As illustrated above, a large sample size is important for accurate reconstruction of past selection, but more fundamentally, it is important that the samples used in the analysis represents the underlying allele frequency curve, i.e., to have data before, during and after the change in allele frequency due to selection. Nevertheless, as also demonstrated in the analyses above, temporal clustering of the data does not necessarily cause biased estimates but will instead result in a wider range of parameter combinations as plausible explanations for the data.

### Selection at the TSHR Locus Coincides with Medieval Changes in Chicken Husbandry

**Fig. 3.**
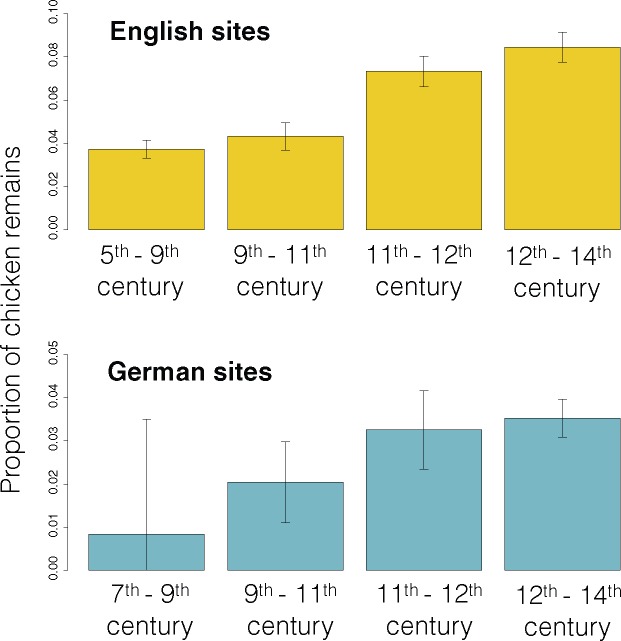
Proportion of chicken remains (by number of identified specimens) from 184 English (top) and 104 German (bottom) archaeofaunal assemblages (Materials and Methods).

Our analysis indicates that selection on the *TSHR* derived allele began around 920 AD (290–1210 AD at 95% level). Intriguingly, this period coincides with major changes in chicken husbandry witnessed in several archaeological assemblages across Northwestern Europe (Boessneck et al. 1979; Heinrich 1987; Pieper and Reichstein 1995; Heinrich 2006; Hüster Plogmann 2006; Serjeantson 2006; Sykes 2007; Holmes 2014). Analyses of data from 184 English archaeological faunal assemblages and 104 German archaeological faunal assemblages demonstrate a substantial increase in the frequency of chicken remains from the Early to the High Middle Ages (see fig. 3 and Materials and Methods) and higher proportions of adult hens in the flocks, presumably to increase egg production (Serjeantson 2006; Sykes 2007; Holmes 2014).

## Discussion

This study demonstrates how inferring allele frequency trajectories through time, using ancient DNA in a flexible Bayesian inference framework, has numerous advantages over previous approaches to studying selection. Firstly, our method provides a means of formally accommodating small sample sizes, uneven temporal distribution of samples and uncertainties in sample ages. Secondly, it allows for episodic selection and gene flow to be explicitly modeled, thus enabling formal integration of independent lines of evidence related to migration, admixture rates, past population sizes and the expression of alleles (dominant or recessive), all of which significantly enhance inferential power. Thirdly, the deterministic nature of our inference approach renders the method fast, flexible, and capable of handling any genotype or allele frequency data from samples before, during and after the hypothesized period of selection. Additionally, as demonstrated by our sensitivity analyses, the approach can also be used to (iteratively) identify the most informative time periods for selection analysis (the periods with the largest uncertainty in the reconstruction of the allele trajectory), and thereby be used as a tool for selecting samples for genetic sequencing.

Although determining the causal factors behind the inferred selection on the TSHR locus is beyond the scope of this paper, by combining ancient DNA with statistical modeling we can increase our ability to identify specific ecological or socio-cultural drivers behind selection. Rather than relying purely on knowledge of the biological function of genes to infer causes of selection, our method allows to also include factors such as known climatic or socio-cultural developments inferred, from geological, archeological or historical records that are broadly contemporaneous with the time of inferred allele frequency changes.

For example, our inferred timing of the onset of selection on the *TSHR* derived allele brackets a key period of changes in dietary preferences and chicken husbandry practices across northwestern Europe between the 9th and 12th centuries AD (fig. 3). The significant intensification of chicken and egg production, evident in the Medieval European archaeological record, has been linked to Christian fasting practices which forbade the consumption of meat from four-legged animals during fasting periods, but allowed for the consumption of birds, eggs, and fish (Venarde 2011). These rules, which originated in the Benedictine Monastic Order, became widely adopted across Europe due to increasing political influence of the Catholic church and applied to all segments of society ca. 1000 AD (Sykes 2007). An increase in chicken consumption has also been observed in Viking sites in present-day Scandinavia and Northern Germany (Boessneck et al. 1979; Pieper and Reichstein 1995; Hüster Plogmann 2006), coinciding with Christianization of these areas (starting in the late 10th–11th and the mid-9th centuries AD, respectively) (Näsman 1976; Sanmark 2004).

Religiously inspired dietary preferences are not the only factors potentially affecting medieval poultry consumption. The observed increase in chicken production could also have been driven by urbanization and population growth (Bartlett 1994), facilitated by the widespread introduction of the more efficient agricultural practices including the three-field system with crop rotation and new tools such as the heavy plough (White 1967; Andersen et al. 2016) as well as favorable climatic conditions (Mann et al. 2009) in the High Middle Ages. Although heavily reliant on the agricultural land surrounding the towns and the cities (Keene 1995), the urban population likely secured some of its own needs by keeping single animals such as goats, pigs and small flocks of chickens in the domestic space in order to have predictable access to milk, eggs, and occasionally meat. These factors could, individually or in combination, explain the observed medieval increase in poultry consumption, which would most likely have resulted in intensified production of poultry. This could have been achieved either through increasing chicken flock sizes or (in an urban setting) flock densities, which could have been facilitated by selection on reduced aggression within the flocks (Karlsson et al. 2015) or faster onset of egg laying at sexual maturity (Karlsson et al. 2016), both demonstrated effects of the derived TSHR allele.

For BCDO2, we also find some support of selection. However, our analysis suggests that the significant rise in allele frequency between ancient and modern noncommercial chicken breeds can be attributed to recent gene flow from Asia. It is also possible that the commercial chicken lines (excluded from this study) have experienced additional strong selection at the BCDO2 locus. This is supported by observation that the frequency of the derived BCDO2 allele is much higher in commercial chicken lines compared to the local chicken breeds used in this study (Rubin et al. 2010).

To our knowledge, the spread of the TSHR derived allele is the first example of a preindustrial domesticate trait selection in response to a historically attested cultural shift in food preference. This result supports the view (Allaby et al. 2008) that domestication is an ongoing process where numerous traits that have been traditionally associated with domestication underwent selection long after the initial domestication phases, and that domestic populations have experienced highly variable selective regimes in response to shifting cultural preferences. More generally, humans and their domestic plants and animals have undergone radical shifts in allele frequencies as they have each responded to alterations in natural and artificial selective pressures over the past 15,000 years. The ability to infer allele frequency trajectories and the timing and strength of selection episodes, based upon time-stamped genetic data, allows for those episodes to be correlated with specific ecological and cultural drivers, especially for drivers that are not present in contemporary populations. This in turn can reveal both the processes and causative mechanisms responsible for generating the patterns of genomic variation in humans and their codependent domestic plants and animals found today.

## Materials and Methods

### Ancient Genotypes

We use genotype data from two loci, TSHR (position 43,250,347 on chromosome 5) and BCDO2 (position 6,273,428 on chromosome 24), both argued to have undergone selection in domestic chickens. The two loci are typed, respectively, in 59 and 30 nonmodern West Eurasian and Moroccan chickens, temporally raging approximately from 2,200 years ago to present day. The genotypes of ancient West Eurasian individuals as well as their approximate ages are taken from Flink et al. (2014). We increased the size of data set for both TSHR and BCDO2 locus by 14 and 4 individuals, respectively. These additional samples were genotyped using the same protocol as reported in Flink et al. (2014) (supplementary Information, Supplementary Material online for details). Six ancient samples (four samples out of the 14 samples newly presented in this study) were directly radiocarbon dated (supplementary table S7, Supplementary Material online). The rest of the samples were dated by stratigraphic association. In order to minimize the possibility of intrusion from more recent periods all indirectly dated samples originate from sites with no following occupational layers (e.g., no building structures or archaeological strata). See supplementary tables S2 and S3, Supplementary Material online for list of all samples used in the analyses, as well as their genotypes at the two loci and ages, for TSHR and BCDO2, respectively.

### Modern Allele Frequencies

The modern allele frequencies for TSHR locus is calculated from the total frequency of selected allele in sampled 167 nonindustrial European chickens, representing 24 breeds. Data presented in (Rubin et al. 2010) (see supplementary table S4, Supplementary Material online for breeds included and the TSHR allele frequencies in those breeds).

As the allele frequencies at the BCDO2 locus in modern populations are unknown we use the phenotypes of 18 modern nonindustrial European chicken breeds, reported in European collaboration project on chicken biodiversity database (AVIANDIV: http://w3.tzv.fal.de/aviandiv; last accessed November 1, 2015) (supplementary table S5, Supplementary Material online) for breeds included, typical phenotypes and estimated average population sizes). As the hypothesized selected BCDO2 allele is recessive, the birds from breeds reported to be yellow legged were considered to carry two copies of the selected allele. For birds from breeds reported to be predominantly white legged, the frequency of the proposed selected allele was assumed to be 0.016, based on the estimates of frequency in the white legged European breeds in (Eriksson et al. 2008) (see supplementary table S6, Supplementary Material online for the breeds included) and assuming Hardy–Weinberg equilibrium.

To factor in the uncertainty rising from the fact that the BCDO2 allele counts are not independent observations but estimates themselves, we used bootstrapping at the level of breeds to estimate the uncertainty in the population wide frequency. We then approximate this distribution with a binomial distribution by fitting the mean and the variance of the binomial distribution to the bootstrapped distribution, yielding an effective sample size of 18.4 individuals. This approximation allows us to give the estimate an equal weight to the ancient samples when calculating the likelihoods in the model.

## Fixed Parameters

### Timing of Gene Flow from Asia

The starting time of gene flow from Asia is historically well documented (Dana et al. 2011; Flink et al. 2014; Lyimo et al. 2015) and as a result, is fixed to start at 250 years ago and lasts until the present.

### Rate of Gene Flow From Asia

The rate of gene flow was estimated using modern and ancient mitochondrial haplogroup frequencies: Based on all samples currently available, all European chickens prior to historically documented gene flow from Asia belonged to mitochondrial haplogroup E (Flink et al. 2014). Today 90% of European chickens belong to haplogroup E while the rest are assigned to haplogroups A, B, and C. Haplogroup E is also most common among Asian chicken populations, however only 30% of the sampled Asian Chickens belong to this haplogroup. Assuming that the change in the frequency of haplogroup E is the direct result of gene flow from Asia and that this gene flow has not been sex biased, we can estimate that ∼15% of the modern European chickens have Asian origin. As a result, the rate of gene flow per year in the model is assumed to be equal to the proportion of European chickens with Asian ancestry (15%) divided by the length of the admixture period (250 years).

### Allele Frequencies in the Source Population

The frequency of the selected allele in the Asian population at the time of the gene flow is unknown. Therefore, we used a conservative estimate of (*f*_external_ = 1), i.e., the selected allele is fixed in Asian chickens. We chose this conservative approach to minimize the possibility of overestimating the role of selection at increasing the allele frequency over the effect of gene flow from Asia. As a result, lower frequency in Asian population during the period of the gene flow could result in later estimates of the starting times of selection and higher estimates of selection coefficients in the two loci considered. However, for TSHR we observe that selection brings the frequency to near fixation before the onset of gene flow, and therefore the precise frequency of the derived allele in Asia is not very important for the allele frequency trajectory. For BCDO2, a lower frequency of the derived allele in Asia could lead to slightly higher levels of selection.

## Model Fitting

In order to infer starting time of selection, selection coefficient, and ancestral frequency of a selected allele, we perform a full sweep of those parameters and calculate the deterministic allele frequency trajectory for each parameter combination. The deterministic allele frequency curves were calculated using *lsoda* function in R package *deSolve* (Soetaert et al. 2010).

### Value Ranges Considered for Estimated Parameters

Parameter values considered for starting time of selection are between 0 to 2,000 years ago in uniformly spaced steps of ten years. For the selection coefficient, we considered 100 uniformly spaced points between 10^−4^ and 1 on a log scale. Parameter values considered for ancestral frequency of a selected allele ranged from 0.01 to 1 in 100 steps. We derive the marginal likelihoods for each of the parameters (starting time of selection, selection coefficient and the starting time of selection) by averaging over the likelihoods of the remaining two parameters.

### Incorporating Sample Age Uncertainty

Following the Bayesian principle, we treat the age of each sample as a random variable with uniform distribution over its age range (see supplementary tables S2 and S3, Supplementary Material online for age ranges), and calculate the posterior likelihood of the sample data given this distribution (numerically approximating the continuous distribution within each interval by ten equally spaced time points)

## Test of Significance

### Stochastic Simulations

In the model described above the change in the allele frequency is assumed to be a result of selection and gene flow, and that the effective population size is large enough to ignore genetic drift. To test if selection is needed to explain the change in the allele frequency through time we explicitly test whether the selection models are statistically different from models including drift and gene flow alone. We do this by simulating allele frequency trajectories with drift, under the assumption of no selection but the same gene flow parameter values as in the deterministic allele frequency trajectories above.

We use the *sde.sim* function in the R package *sde* (Iacus 2008) to simulate 10,000 allele frequency trajectories using the stochastic differential equation corresponding to equation (1) without selection and including genetic drift in a population of size *N*_e_:

(Eq. 3) $$\frac{df}{dt}=m\left(t\right)\left(f_{\text{external}}-f\right)+\sqrt{f\left(1-f\right)/2N_{e}}\xi (t),$$

To match the selection model described above, ancestral allele frequency is sampled from a uniform distribution over the interval [0,1]. The population size *N*_e_ is sampled from the posterior distribution of population size estimates from BEAST (Drummond et al. 2012) (see below), with a median value of 1.8*10^5^ and 95% HPD interval ranging from 26,000 to 4.6*10^5^. Since this population size estimate is based on a nonrecombining and maternally inherited locus, the total effective population size should be four times that. However, as male to female ratio in ancient European chicken population is unknown, we take a conservative approach and use the minimum effective population size, which roughly corresponds to the size of the female effective population size. Therefore, we multiply the posterior distribution of population size by factor of two to get an estimate of a minimum effective population size. This approach is conservative since the power of genetic drift to influence allele frequencies decreases with increasing population size. To formally evaluate whether the model with selection provides a significantly better fit for the data, we calculate the proportion of log likelihood values from the stochastic simulations that are as high or higher than the log likelihood of the selection model.

### Chicken Population Size Estimation Using BEAST

We estimated an order of magnitude effective population size for chickens through time using Bayesian Phylogenetic tool BEAST (Drummond et al. 2012) and a 200 base pairs long mitochondrial control region fragment from 194 modern European chickens and 39 ancient European individuals with known ages and a uniform prior for constant population size ranging [0, 10^−7^], (for the full BEAST input file see supplementary File S1, Supplementary Material online).

## Sensitivity Analyses

### Hardy–Weinberg Analysis

We selected samples dating to before 300 AD to test for deviation from Hardy–Weinberg equilibrium. Due to the large sample size (*n* = 37 for TSHR and *n* = 24 for BCD02) we used the standard χ^2^ test (with one degree of freedom) to investigate whether the genotype frequencies are binomially distributed.

### North-Western Subset of the Data (Sensitivity to Potential Geographic Structure)

We generated a reduced data set covering North-West Europe by excluding ancient samples from Turkey, Greece, and Morocco, resulting in 42 ancient samples for TSHR locus and 30 ancient samples for BCDO2 locus (supplementary fig. S4, Supplementary Material online) for the temporal distribution of these samples). For consistency, all data points from Italy and Spain (BCDO2) and Italy and Israel (TSHR) were also excluded from the calculation of modern allele frequencies (but this did not affect the modern frequencies).

### Simulated Data (Sensitivity to Sample Size)

We chose a combination of parameters similar to that found for TSHR (starting time of selection = 1000 AD, selection coefficient = 0.005 and ancestral frequency = 0.4). For simplicity, we assume no gene flow from Asia (*m* = 0). We simulated ten replicate data sets each of 10, 20, 50, 100, and 200 ancient diploid samples (and 150 modern samples). Simulated ancient samples were given randomly chosen dates (uniformly distributed from year 0 to 2000 AD) and 100 years age uncertainty (on both sides of the assigned age). For each individual, we generated random diploid genotypes according to the derived allele frequency given by the model at the sample age.

## Archaeological Chicken Remains Frequency Calculation

We used data from Appendix Ia in Sykes (2007) on the faunal number of identified specimens (NISP) from 184 English archaeological faunal assemblages and compiled information (supplementary File S2, Supplementary Material online) on faunal NISP from 104 German archaeological faunal assemblages to estimate the relative frequency of chicken remains at each of the four time periods for English and German sites, respectively. We calculated the relative frequency by dividing the number of identified chicken specimens by the total faunal NISP for the same time period (fig. 3 and supplementary table S1, Supplementary Material online) The 95% confidence intervals were calculated using the normal distribution approximation (justified by the large sample sizes).

The framework described above is implemented in the statistical environment R. The R code is available to download from GitHub (https://github.com/LiisaLoog/Selection-Framework) and from L.L upon request.

## Supplementary Material

Supplementary data are available at *Molecular Biology and Evolution* online.

## Supplementary Material

Supplementary Data
