# Supplementary material for: Inferring Allele Frequency Trajectories from Ancient DNA Indicates That Selection on a Chicken Gene Coincided with Changes in Medieval Husbandry Practices
Source: Mol Biol Evol. 2017 May 2;34(8):1981–90. doi: 10.1093/molbev/msx142 (PMC5850110; doi:10.1093/molbev/msx142)
Supplement: Supplementary Data [file msx142_Supp.zip › Loog.et.al.2017_SI.pdf]

Supplementary Information for Loog et al. (2017) "Inferring allele frequency trajectories from ancient DNA indicates that selection on a chicken gene coincided with changes in medieval husbandry practices"

WET LABORATORY PROCEDURES AND PROTOCOLS 2

ANCIENT DNA LABORATORIES AND EXPERIMENTAL SET-UP 2
ANCIENT DNA EXTRACTION 2
DNA GENOTYPING 3

ARCHEOLOGICAL BACKGROUND FOR NEWLY GENOTYPED SAMPLES 4

MOGADOR 4
DOLICHE/DÜLÜK BABA TEPES 4
KORUCUTEPE/ELAZIG 4

SUPPLEMENTARY FIGURES 5

SUPPLEMENTARY FIGURE 1 5
SUPPLEMENTARY FIGURE 2 6
SUPPLEMENTARY FIGURE 3 7
SUPPLEMENTARY FIGURE 4 8
SUPPLEMENTARY FIGURE 5 9
SUPPLEMENTARY FIGURE 6 10
SUPPLEMENTARY FIGURE 7 11
SUPPLEMENTARY FIGURE 8 12

SUPPLEMENTARY TABLES 13

SUPPLEMENTARY TABLE 1A 13
SUPPLEMENTARY TABLE 1B 13
SUPPLEMENTARY TABLE 2 13
SUPPLEMENTARY TABLE 4 16
SUPPLEMENTARY TABLE 5 17
SUPPLEMENTARY TABLE 6 17
SUPPLEMENTARY TABLE 7 18
SUPPLEMENTARY TABLE 8 18
SUPPLEMENTARY TABLE 9A 18
SUPPLEMENTARY TABLE 9B 18

REFERENCES 19

Wet Laboratory Procedures and Protocols

Ancient DNA Laboratories and Experimental Set-Up

DNA extractions and PCR amplifications were performed in a dedicated ancient DNA laboratory in the Department of Archaeology (Durham Evolution and Ancient DNA) at Durham University, United Kingdom. We followed strict laboratory procedures according commonly used guidelines (Cooper and Poinar 2000; Gilbert et al. 2005). All equipment and work surfaces were cleaned before and after each use with a dilute solution of bleach [5–10% (wt/vol) active sodium hypochlorite] followed by ddH2O and ethanol [99% (vol/vol)]. Pipettes and plastic racks were UV-irradiated in a dedicated cross-linker (at <15 cm for at least 30 min at 254-nm wavelength) before and after use. Pre- and post-PCR laboratories are physically isolated; access to the pre-PCR laboratories is restricted to Ancient DNA laboratory users only and access is also prohibited if the laboratory user had entered post-PCR areas the same day. Ancient DNA laboratory users wear clean laboratory coats, double layer of gloves (nitrile and latex), and overshoes to avoid introducing contaminants from post-PCR areas.

Ancient DNA Extraction

The ancient chicken bones were prepared for DNA extraction by removing an approximately 1-mm layer of outer bone surface by abrasion using a Dremel drill with clean, single-use cut-off wheels (Dremel no. 409). A subsection of the bone was subsequently isolated and pulverized in a Micro-Dismembrator (Sartorious-Stedim Biotech), followed by collection in 15-mL Grainer tubes. Milling containers and grinding balls were subsequently suspended and cleaned in 1% virkon, and rinsed in absolute [99% (vol/vol)] ethanol.

Next, 50- to 100-mg bone powder/specimen was digested in 0.425M EDTA (pH 8), 0.05% SDS, 0.05M Tris·HCI and 400 μg Proteinase K, and incubated overnight on a rotator at 50 °C until fully dissolved. Once dissolved overnight, 2 mL of solution was concentrated in a Millipore Amicon Ultra-4 30 kDa molecular weight cut-off to a final volume of 100 μL. The concentrated DNA extract was purified using the QIAquick PCR Purification Kit (Qiagen) following manufacturers recommendations, except that the final elution step was performed twice (2 × 50 μL) to produce a final volume of 100 μL. Approximately 1 in 10 DNA extractions were blank negative controls containing only extraction buffer and Proteinase K.

PCR Amplification. PCR set-up was performed in a fume hood in a dedicated facility adjacent to the dedicated ancient DNA extraction facility. The PCR set-up facility is subject to positive air pressure that minimizes the risk of introducing contaminant DNA. Approximately 1 in 11 PCR reactions were negative controls.

BCDO2: PCRs were set up in 25-μL reactions using 1.0–1.25 U Taq GOLD (Applied Biosystems), 1× Gold buffer (Applied Bio- systems), 2.5 mM MgCl2, 0.5

µg/µL BSA, 1 M betaine, 200 µM of each dNTP, 0.4 µM of each primer, and 2–5 µL of ancient DNA extract. PCR cycling conditions were 95 °C for 5 min, 50 cycles of 94°C for 30 s, 57 °C for 30 s, and 72 °C for 30 s, followed by 72 °C for 10 min.
TSHR: PCRs were set up in 25 µL reactions using 1.0–1.25 U Taq GOLD (Applied Biosystems), 1× Gold buffer (Applied Bio- systems), 2.5 mM MgCl2, 0.5 µg/µL BSA, 200 µM of each dNTP, 0.6 µM of the biotinylated forward primer, and 0.8 µM of the reverse primer, and 2–5 µL of aDNA extract. PCR cycling conditions were 95 °C for 5 min, 50 cycles of 94 °C for 30 s, 56 °C for 30 s, and 72 °C for 30 s, followed by 72 °C for 10 min. PCRs were visualized on a 1–2% (wt/vol) agarose gel using GelRed and UV illumi- nation. PCR products were purified using ExoSAP-IT (USB Affymetrix) and stored at −20 °C before sequencing.

DNA Genotyping

Pyrosequencing was performed in-house at the Archaeology department in Durham using the PyroMark Q24 (Qiagen) following manufacturers guidelines, and using Qiagen Q24 sequencing reagent kits. Results, sequences, and genotypes were analyzed in the PyroMark Q24 software (Qiagen) using modified settings: accepted peak deviation and minimum peak heights were set to less strict to account for low signal intensity and slight deviations in peak heights (which, if observed, could be the result of Type-2 C→U deamination/error). Dispensation order was automatically generated using the PyroMark Q24 software (Qiagen) (Table S8).
To account for allelic dropout that is common in ancient DNA studies (Svensson et al. 2007), each SNP/genotype was confirmed by repeated genotyping from two to eight independent PCRs (at least two independent replications for heterozygous specimens, but up to eight replications in for homozygous specimens and heterozygous specimens for which we repeatedly observed allelic drop- out). The probability of falsely assigning a heterozygous individual as homozygous was calculated as follows: P(false homozygote) = K × (K/2)n − 1, where n is the number of replicates and K is the observed number of allelic dropouts divided by the total number of genotypings of heterozygous individuals (Gagneux et al. 1997; Svensson et al. 2007)

Archeological background for newly genotyped samples

The 14 new samples come from three different archaeological sites located in Morocco and Turkey.

Mogador

Four samples (RB579; RB585; RB585; RB587) come from the site of Île de Mogador in Morocco. The site has three main occupational periods: The first corresponds to a Phoenician inhabitation between the 7th and 5th century BC. The second major occupation phase dates to the Roman period and lasted from the 1st till the 3rd century CE. The chicken samples newly genotyped for this study come from a third period, corresponding to a modern re-occupation, which started in the 17th/18th century and lasted into the 20th century CE. The chicken samples, newly genotyped for this study, are dated to be not older than 50 years (Table S7). General information about the site together with a detailed study of its fauna is available in Becker et al. (2012).

Doliche/Dülük Baba Tepes

Three samples (RB593; RB594; RB595) come from a hill-top site of Doliche/Dülük Baba Tepesi near modern Gaziantep in Southern Anatolia, Turkey. The site, famous for being the place where the cult of Iupiter Dolichenus originated, was occupied from the 6th century BC until the 13th century AD. In modern times (until recently) the site witnessed civilian and military use. The chicken samples, newly genotyped for this study, come from modern refuse overlying antique occupation and are dated to be not older than 50 years (Table S7). Archaeological background of the site and a detailed report containing information about the faunal assemblage can be found in Pöllath and Peters (2011).

Korucutepe/Elazig

Seven samples (RB598; RB599; RB600; RB601; RB602; RA26; RA27) come from the site on Korucutepe/Elâzığ located in Eastern Anatolia, Turkey. Site occupation started in the second half of the 4th millennium BC and continued during the Bronze and early Iron Ages. Around 800 BC the site was abandoned until its resettlement by Seljuqs some two thousand years later (between 1200 and 1400 AD). After this time period occupation ceased at the site. Samples genotyped for this study have been dated to be around 700 years old (Table S7), thus coinciding with the Seljuk occupation. General information about the site can be found in Boessneck and von den Driesch (1975).

Supplementary Figures

Supplementary Figure 1

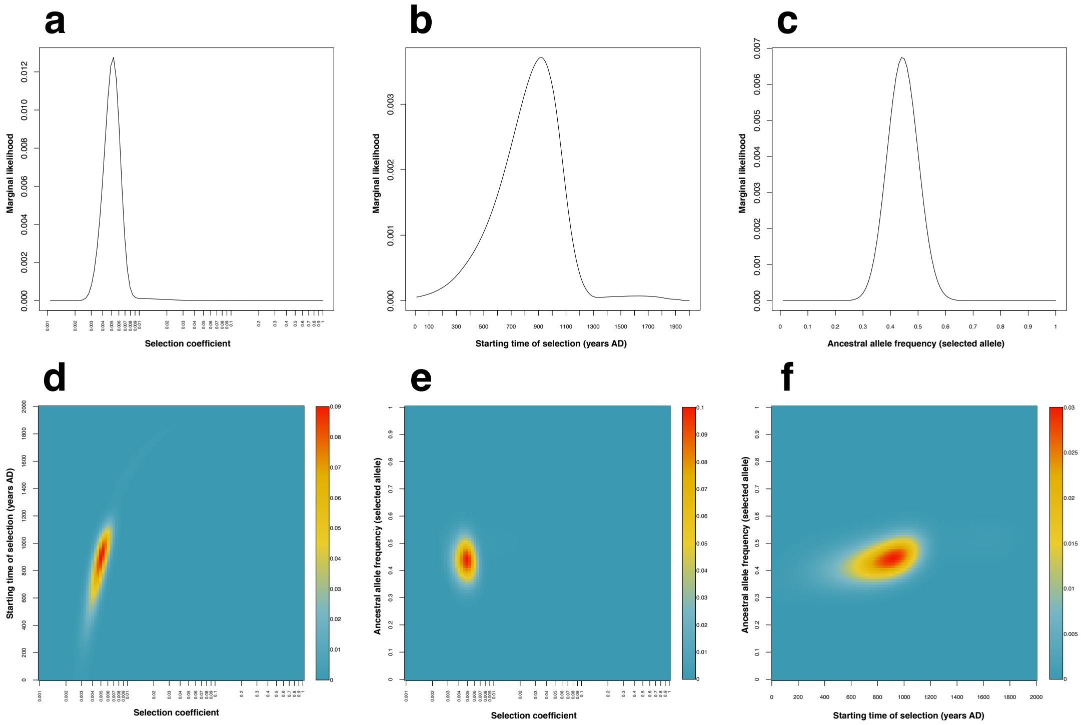

Figure S1. Marginal posterior distribution of parameters for the TSHR locus: selection coefficient (**a**), starting time of selection (**b**) and ancestral allele frequency (**c**). Panels **d**-**f** show joint marginal posterior distribution of pairs of parameters.

Supplementary Figure 2

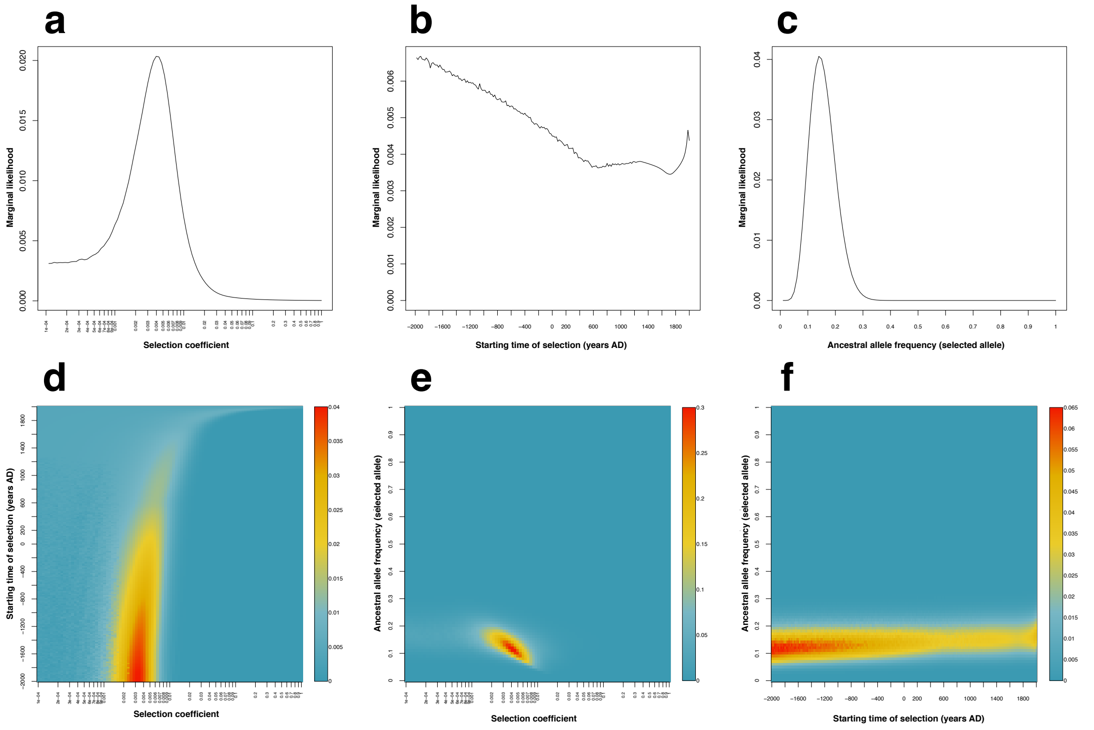

Figure S2. Marginal posterior distribution of parameters for the BCDO2 locus: selection coefficient (**a**), starting time of selection (**b**) and ancestral allele frequency (**c**). Panels **d**-**f** show the joint marginal posterior distribution of pairs of parameters.

Supplementary Figure 3

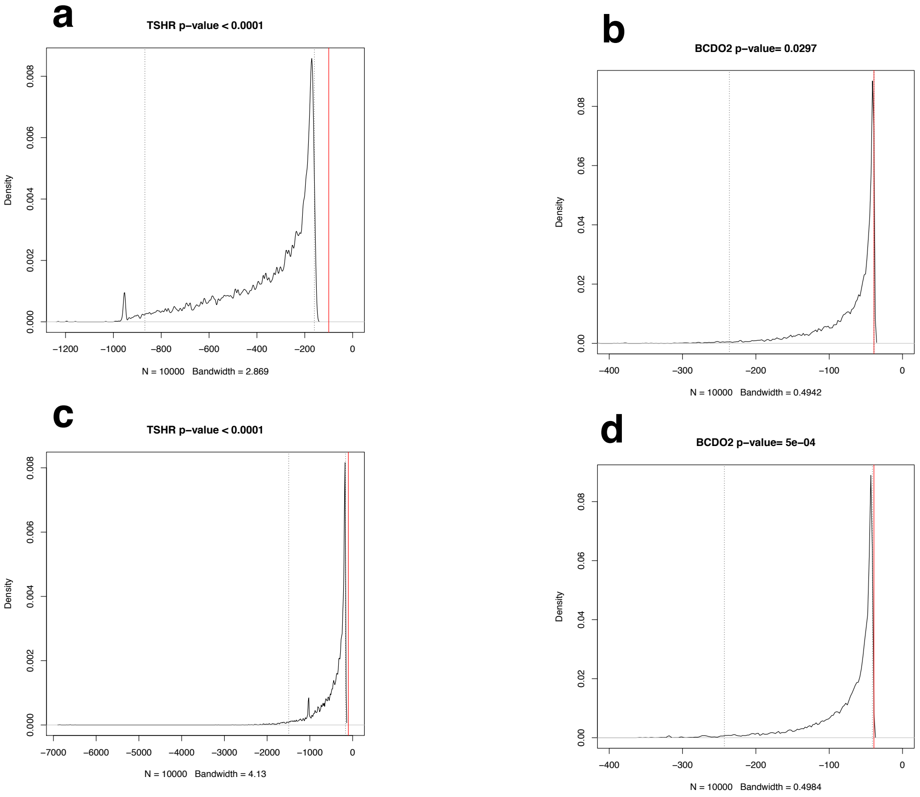

Figure S3. Distribution of data likelihoods for the TSHR locus from 10,000 simulations of random genetic drift with (**a**) and without (**c**) gene flow from imported Asian jungle fowl. Panels **b** and **d** show the corresponding results for the BCDO2 locus.

Supplementary Figure 4

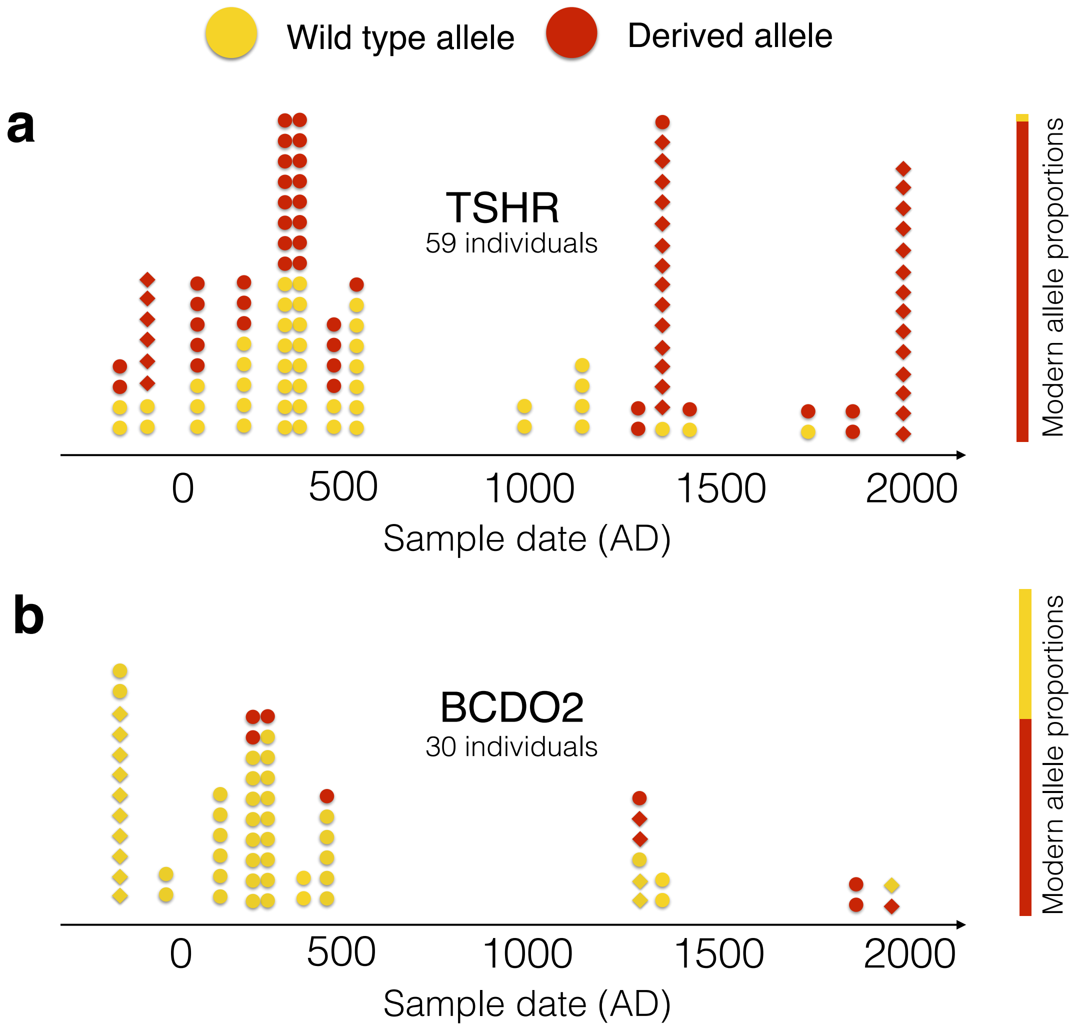

Figure S4: Observed allele counts through time for the TSHR locus (**a**) and the BCDO2 locus (**b**). Wild type alleles in ancient samples are represented by yellow dots and derived alleles by red dots. Modern allele proportions are shown as solid bars to the right of each panel. Circles represent ancient samples from North-Western Europe and diamonds represent ancient samples from the Mediterranean region.

Supplementary Figure 5

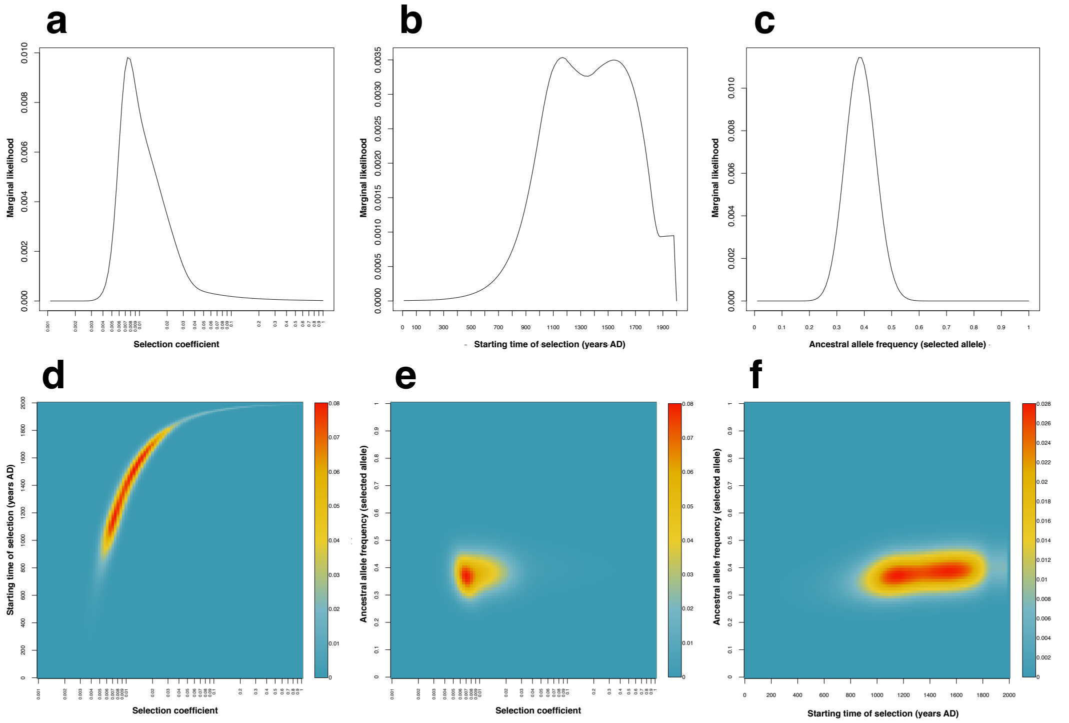

Figure S5. Marginal posterior distribution of parameters for the TSHR locus in the North-Western Europe subset of the data: selection coefficient (**a**), starting time of selection (**b**) and ancestral allele frequency (**c**). Panels **d**-**f** show joint marginal posterior distribution of pairs of parameters.

Supplementary Figure 6

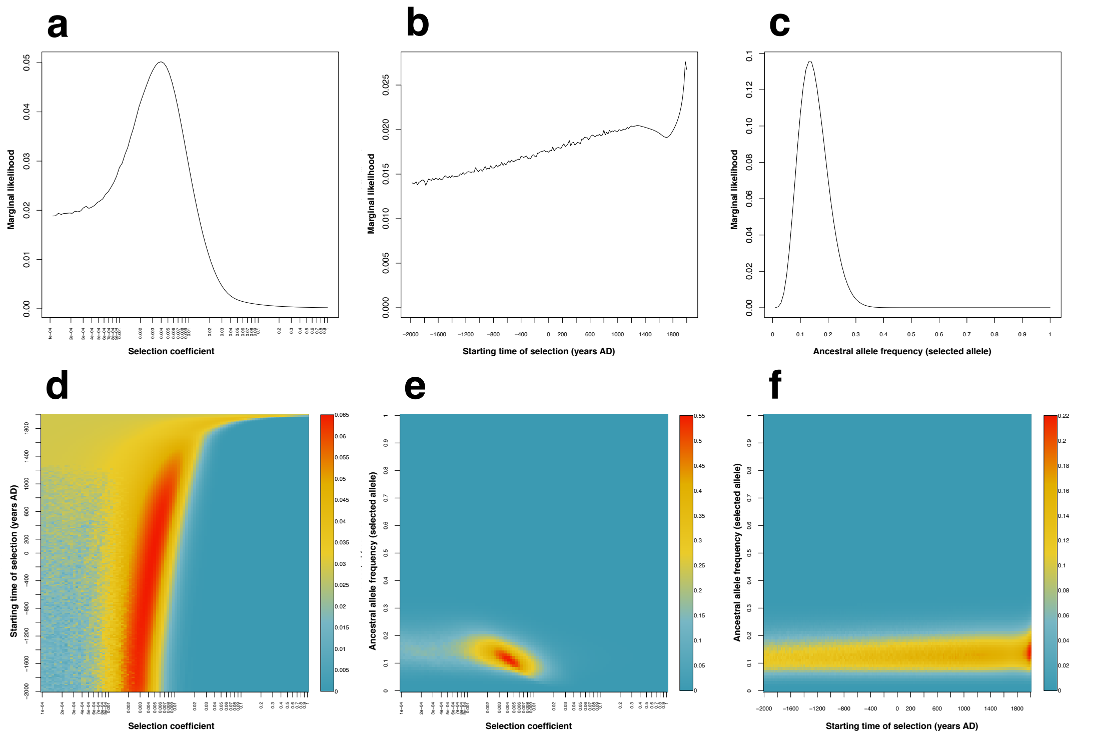

Figure S6. Marginal posterior distribution of parameters for the BCDO2 locus in the North-Western Europe subset of the data: selection coefficient (**a**), starting time of selection (**b**) and ancestral allele frequency (**c**). Panels **d**-**f** show the joint marginal posterior distribution of pairs of parameters.

Supplementary Figure 7

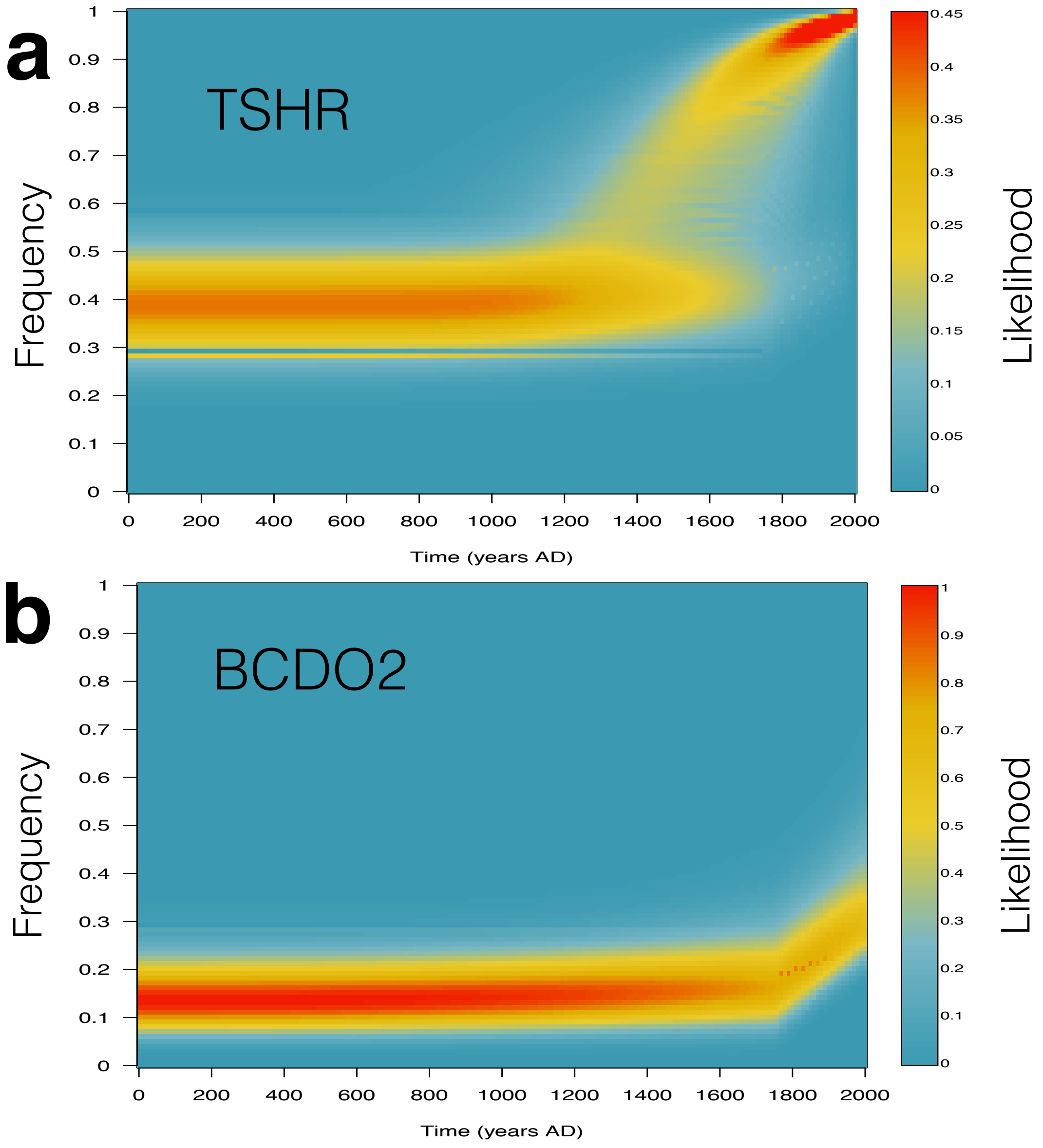

Figure S7: Posterior distribution of the derived allele frequency as a function of time for the TSHR locus (a) and the BCDO2 locus (b) in the North Western Europe subset of the data. The likelihoods are color coded (see color bar) and are shown relative to the maximum likelihood in the plot.

Supplementary Figure 8

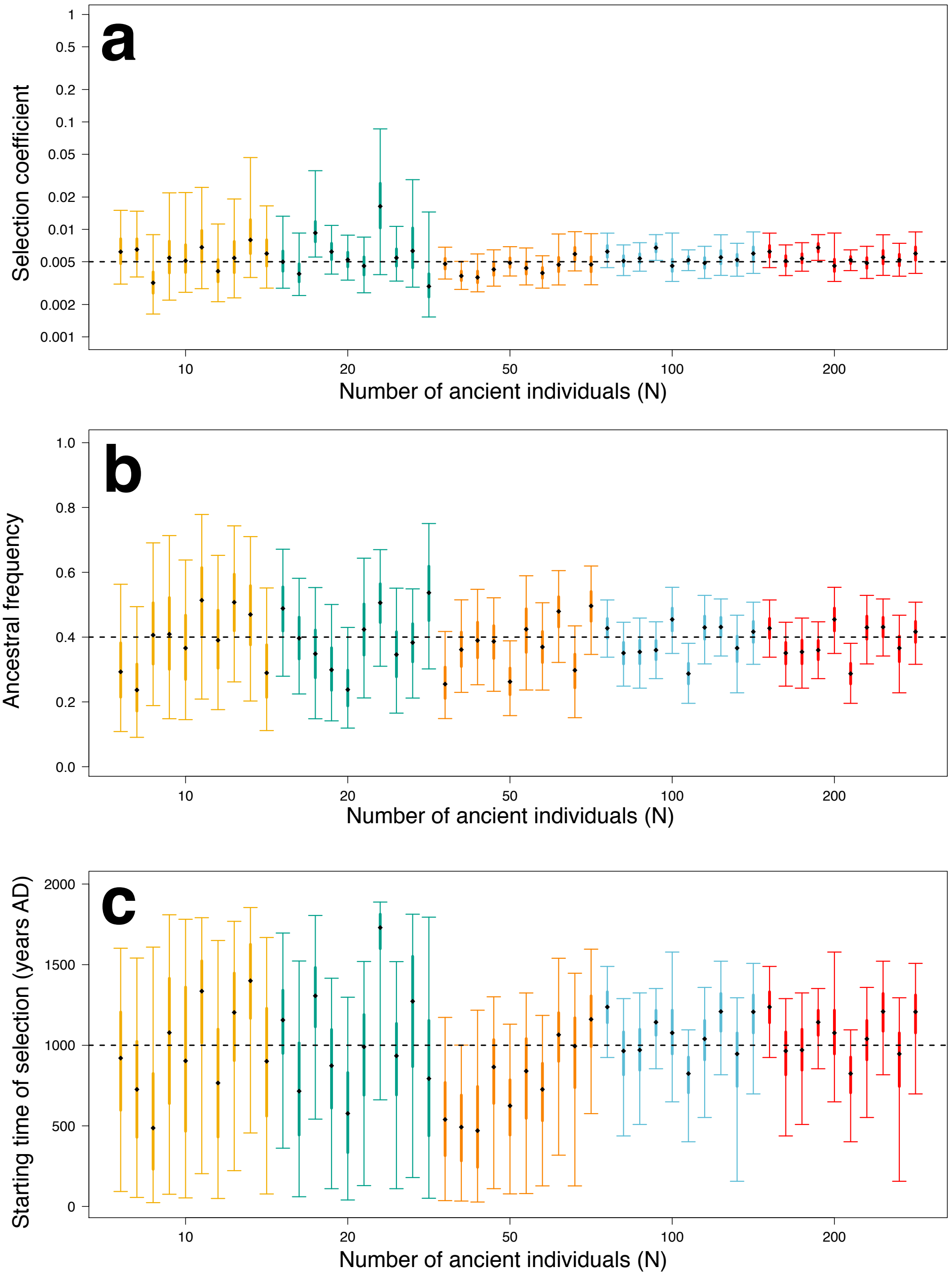

Figure S8: Effect of sample size on the parameter estimates in simulated data. Marginal estimated parameter distributions for selection coefficient (**a**), ancestral derived allele frequency (**b**) and starting time of selection (**c**, in years AD). For each simulated dataset, we show the median (black dot), inter-quartile range (thick line) and symmetric 95% confidence intervals (thin line). Data sets are grouped according to number of simulated ancient individuals (*N*) and coloured by size: *N*=10 (yellow), *N*=20 (green), *N*=50 (orange), *N*=100 (blue) and *N*=200 (red).

Supplementary Tables

Supplementary Table 1a

Period (century AD)	Total number of chicken remains	Total number animal remains	Proportion of chicken remains	95% Confidence Intervals	
5th to 9th	8029	215655	0.037230762	0.04137207	0.03308946
9th to 11th	3856	89374	0.043144539	0.04955772	0.03673136
11th to 12th	5237	71435	0.073311402	0.0803708	0.06625201
12th to 14th	6228	73729	0.084471511	0.09137825	0.07756478

Supplementary Table 1b

Period (century AD)	Total number of chicken remains	Total number animal remains	Proportion of chicken remains	95% Confidence Intervals	
7th to 9th	45	5364	0.008389262	-0.01825982	0.03503835
9th to 11th	866	42353	0.020447194	0.01102118	0.0298732
11th to 12th	1456	44713	0.032563237	0.02344627	0.04168021
12th to 14th	6754	191586	0.035253098	0.03085484	0.03965136

Table S1. Relative frequency of domestic chicken remains (by number of identified specimens) from 184 English archaeological faunal assemblages (Table S1a) and 104 German archaeological faunal assemblages (Table S1b).

Supplementary Table 2

ID	Country	Genotype	From (Years AD)	To (Years AD)	Dating Method	Reference
Ch37	Germany	GA	-280	-15	Context	Flink et al. 2014
Ch40	Germany	GA	-280	-15	Context	Flink et al. 2014
RA6	Greece	AA	-200	-40	Context	Flink et al. 2014
RA18	Greece	AA	-200	-40	Context	Flink et al. 2014
RA22	Greece	AA	-200	-40	Direct	Flink et al. 2014
Ch31	Germany	GG	-200	-30	Context	Flink et al. 2014
Ch33	Austria	GA	-100	50	Context	Flink et al. 2014
Ch34	Austria	GA	-100	50	Context	Flink et al. 2014
Ch35	Austria	AA	-100	50	Context	Flink et al. 2014
Ch36	Austria	GA	-100	50	Context	Flink et al. 2014
RB373	England	GG	69	96	Context	Flink et al. 2014
RB374	England	GA	69	96	Context	Flink et al. 2014
RB375	England	GA	69	96	Context	Flink et al. 2014
RB376	England	GA	69	96	Context	Flink et al. 2014
Ch3	Germany	GG	100	300	Context	Flink et al. 2014
Ch4	Germany	AA	100	300	Context	Flink et al. 2014
Ch7	Germany	AA	100	300	Context	Flink et al. 2014
Ch10	Germany	GG	100	300	Context	Flink et al. 2014
Ch12	Germany	GA	100	300	Context	Flink et al. 2014
Ch13	Germany	GA	100	300	Context	Flink et al. 2014
Ch14	Germany	GG	100	300	Context	Flink et al. 2014
Ch15	Germany	GA	100	300	Context	Flink et al. 2014

Ch16	Germany	GA	100	300	Context	Flink et al. 2014
Ch17	Germany	GA	100	300	Context	Flink et al. 2014
Ch19	Germany	AA	100	300	Context	Flink et al. 2014
Ch20	Germany	GA	100	300	Context	Flink et al. 2014
Ch21	Germany	GG	100	300	Context	Flink et al. 2014
Ch22	Germany	GA	100	300	Context	Flink et al. 2014
Ch23	Germany	GG	100	300	Context	Flink et al. 2014
Ch24	Germany	GA	100	300	Context	Flink et al. 2014
Ch25	Germany	GA	0	500	Context	Flink et al. 2014
Ch26	Germany	GG	0	500	Context	Flink et al. 2014
Ch27	Germany	GA	0	500	Context	Flink et al. 2014
RB368	England	GG	120	400	Context	Flink et al. 2014
RB369	England	GG	120	400	Context	Flink et al. 2014
RB370	England	GG	120	400	Context	Flink et al. 2014
RB372	England	GA	120	400	Context	Flink et al. 2014
RB383	England	GG	900	1100	Context	Flink et al. 2014
RB378	England	GG	1000	1200	Context	Flink et al. 2014
RB381	England	GG	1000	1200	Context	Flink et al. 2014
RB384	England	AA	1100	1400	Context	Flink et al. 2014
RB388	England	GA	1100	1500	Context	Flink et al. 2014
RB599	Turkey	AA	1272	1334	Direct	This study
RB600	Turkey	AA	1275	1337	Context	This study
RB601	Turkey	AA	1275	1337	Context	This study
RB602	Turkey	AA	1275	1337	Context	This study
RA26	Turkey	AA	1275	1337	Context	This study
RA27	Turkey	AA	1275	1337	Context	This study
RB598	Turkey	AA	1278	1340	Direct	This study
RB380	England	GA	1300	1500	Context	Flink et al. 2014
RB385	England	GA	1500	1800	Context	Flink et al. 2014
Ch38	Germany	AA	1820	1880	Direct	Flink et al. 2014
RB579	Morocco	AA	1950	2000	Direct	This study
RB585	Morocco	AA	1950	2000	Context	This study
RB586	Morocco	AA	1950	2000	Direct	This study
RB587	Morocco	AA	1950	2000	Context	This study
RB593	Turkey	AA	1950	2000	Context	This study
RB594	Turkey	AA	1950	2000	Context	This study
RB595	Turkey	AA	1950	2000	Context	This study

Table S2. List of ancient samples used in the analysis of the TSHR locus (position 43,250,347 on chromosome 5). G is the ancestral allele and A is the derived allele.

Supplementary Table 3

ID	Country	Genotype	From (Years AD)	To (Years AD)	Dating Method	Reference
RA6	Greece	AA	-200	-40	Context	Flink et al. 2014
RA11	Greece	AA	-200	-40	Context	Flink et al. 2014
RA14	Greece	AA	-200	-40	Context	Flink et al. 2014
RA18	Greece	AA	-200	-40	Context	Flink et al. 2014
RA22	Greece	AA	-200	-40	Context	Flink et al. 2014
Ch31	Germany	AA	-200	-30	Context	Flink et al. 2014
Ch36	Austria	AA	-100	50	Context	Flink et al. 2014
RB374	England	GA	69	96	Context	Flink et al. 2014
RB375	England	AA	69	96	Context	Flink et al. 2014
RB376	England	AA	69	96	Context	Flink et al. 2014
Ch3	Germany	GA	100	300	Context	Flink et al. 2014
Ch4	Germany	AA	100	300	Context	Flink et al. 2014
Ch10	Germany	AA	100	300	Context	Flink et al. 2014
Ch12	Germany	GA	100	300	Context	Flink et al. 2014
Ch13	Germany	AA	100	300	Context	Flink et al. 2014
Ch15	Germany	AA	100	300	Context	Flink et al. 2014
Ch16	Germany	AA	100	300	Context	Flink et al. 2014
Ch19	Germany	GA	100	300	Context	Flink et al. 2014
Ch20	Germany	AA	100	300	Context	Flink et al. 2014
Ch21	Germany	AA	100	300	Context	Flink et al. 2014
Ch27	Germany	AA	0	500	Context	Flink et al. 2014
RB368	England	GA	120	400	Context	Flink et al. 2014
RB369	England	AA	120	400	Context	Flink et al. 2014
RB370	England	AA	120	400	Context	Flink et al. 2014
RA26	Turkey	AA	1275	1337	Context	This study
RA27	Turkey	GG	1275	1337	Context	This study
RB598	Turkey	GA	1278	1340	Context	This study
RB380	England	AA	1300	1500	Context	Flink et al. 2014
Ch38	Germany	GG	1820	1880	Context	Flink et al. 2014
RB579	Morocco	GA	1950	2000	Context	This study

Table S3. List of ancient samples used in the analysis of the BCDO2 locus (position 6,273,428 on chromosome 24). A is the ancestral allele and G is the derived allele.

Supplementary Table 4

Breed	Origin	Samples in pool	Frequency
White Leghorn Line 13 (WL-A)	Sweden	11	1
Rhode Island Red	Netherlands	10	1
Rhode Island Red (RIR)	France	8	1
Jaärhöns	Norway	8	1
Poltava clay	Ukraine	8	1
Dorking	UK	3	0.67
Broiler Dam Line B (CB-2	France	9	1
Broiler Sire Line B	France	10	1
Bourbonnaise	France	8	1
Coucou de Rennes	France	7	1
Czech Golden Pencilled	Czech Republic	5	1
Finnish Landrace	Finland	5	1
Friesian fowl	Netherlands	5	1
Houdan	France	5	1
Marans	France	8	1
Owl-bearded	Netherlands	8	1
Red Villafranquina	Spain	5	1
Transsylvanian Naked Neck	Hungary	5	1
Westfa_lischer Totleger	Germany	3	0.67
Yurlov crower	Russia	4	1
Icelandic	Iceland	12	0.92
Inhibition of Gold	France	3	1
Coucou du Vercors	France	5	1
SASSO	France	12	1
**Total nr of individuals:**	**167**	**Total frequency:**	**0.98**

Table S4. Frequency of the TSHR derived allele (position 43,250,347 on chromosome 5) in modern European breeds. (Data from (Rubin et al. 2010)

Supplementary Table 5

Breed	Area of breed origin	Sampling country	Skin colour
Bedouin	Middle east	Israel	White
Owl-bearded (Uilenbaarder)	Netherlands	Netherlands	White
Friesian fowl	Netherlands	Netherlands	White
Bresse noire	France	France	White
Houdan	France	France	White
Marans	France	France	White
Dorking	England	England	White
Finnish Landrace	Finland	Finland	White
Sicilienne Buttercup	Italia	Italia	White
Yurlov crower, Russia	Russia	Russia	White
Westfaeliche Totleger	Germany	Germany	White
Sundheimer	Germany	Germany	Yellow
Old Scand. Ref. Pop	Scandinavia	n/a	Yellow
Green-legged Partridge	Poland	Poland	Yellow
Orlov	Russia	Russia	Yellow
Ukrainian bearded	Ukraine	Ukraine	Yellow
Poltava clay	Ukraine	Ukraine	Yellow
Yurlov crower,Ukraine	Russia	Russia	Yellow

Table S5. List of modern European breeds included for BCDO2 derived allele frequency calculation. (Data from AVIANDIV (https://aviandiv.tzv.fal.de) database)

Supplementary Table 6

Breed	Origin	Breed phenotype	Sample size	Frequency of the derived allele in breeds
Friesian Fowl	Netherlands	white skin	6	0
Padova	Italia	white skin	4	0
Westfälischer Totleger	Germany	white skin	3	0
Houdan	France	white skin	5	0
Dorking	England	white skin	4	0
Red Villafranquina	Spain	white skin	5	0.1
Czech Golden Pencilled	Czech Republic	white skin	5	0
	**Total:**	**32**	**Total frequency:**	**0.015625**

Table S6. Frequency of BCDO2 derived allele (position 6,273,428 on chromosome 24) in modern European breeds. (Data from (Eriksson et al. 2008)

Supplementary Table 7

Sample_ID	Reference	Lab_code	Radiocarbon_Age(BP)	
RB599	This study	OxA-X-2504	754	± 27
RB598	This study	OxA-27436	738	± 24
Ch38	Flink et al.2014	Beta-356195	80	± 30
RB579	This study	OxA-27588	1.12172	± 0.00631
RB586	This study	OxA-27435	1.28372	± 0.00326
RA22	Flink et al.2014	Beta-351382	2030	± 30

Table S7. Radiocarbon ages (BP) for directly dated samples used in the analyses.

Supplementary Table 8

Locus	Primer sequence (5'–3')	Primer name	Source	Sequence to analyze
TSHR	CTTTCTTCTTGCCCTTTT	TSHR-F (biotin)	Flink et al. 2014	
TSHR	GATGCTGACTTTGCTGTA	TSHR-R	Flink et al. 2014	
TSHR	TGCTGTAGCTGCTGACTC	TSHR-S	Flink et al. 2014	C/TAACCAGTGG
BCDO2	ACTCTTGCATGGATCTGG	BCDO2-F (biotin)	Flink et al. 2014	
BCDO2	TGTGGTCTCAGAATTTGG	BCDO2-R	Flink et al. 2014	
BCDO2	TCAGAATTTGGGACG	BCDO2-S	Flink et al. 2014	C/TTGGCAATGC

Table S8. Polymerase Chain Reaction and sequencing primers used for TSHR and BCDO2

Supplementary Table 9a

BCD02	s	f.anc	TSHR	s	t.sel.start	f.anc
CI.lower (95%)	0.000131826	0.07	CI.lower(0.025)	0.003019952	290	0.34
CI.upper (95%)	0.01584893	0.25	CI.upper(0.975)	0.00691831	1210	0.54
Max	0.003630781	0.14	Max	0.004897788	920	0.44

Supplementary Table 9b

BCD02	s	f.anc	TSHR	s	t.sel.start	f.anc
CI.lower (95%)	0.000120226	0.06	CI.lower(0.025)	0.004570882	750	0.28
CI.upper(95%)	0.02089296	0.25	CI.upper(0.975)	0.05495409	1900	0.49
Max	0.003630781	0.13	Max	0.00691831	1170	0.38

Table S9. Marginal likelihoods for the selection coefficient (s); ancestral allele frequency (f.anc) and starting time of selection (t.sel.start) estimated for the TSHR and BCDO2 loci. Table 9a contains the estimates and the 95% confidence intervals (CIs) from the analysis with the full dataset. Table 9b contains the estimates and the confidence intervals (CIs, calculated at the 0.025 and 0.975 quintiles))from the analysis with the subset of full dataset containing samples from North Western Europe Only.

References

Becker C, von den Driesch A, Küchelmann HC. 2012. Mogador, eine Handelsstation am westlichen Rand der phönizischen und römischen Welt - die Tierreste. In: Grupe G, McGlynn G, Peters J, editors. Documenta Archaeobiologiae. 10. Rahden/Westfalen: Leidorf. p. 11–160.

Boessneck J, von den Driesch A. 1975. Tierknochenfunde vom Korucutepe bei Elâzığ in Ostanatolien (Fundmaterial der Grabungen 1968 und 1969). In: van Loon MN, editor. Korucutepe. Vol. 1. Amsterdam/Oxford/New York: North-Holland Publishing Company and American Elsevier Publishing Company. p. 1–220.

Cooper A, Poinar HN. 2000. Ancient DNA: Do It Right or Not at All. Science 289:1139–1139.

Eriksson J, Larson G, Gunnarsson U, Bed'hom B, Tixier-Boichard M, Strömstedt L, Wright D, Jungerius A, Vereijken A, Randi E, et al. 2008. Identification of the Yellow Skin Gene Reveals a Hybrid Origin of the Domestic Chicken. PLoS Genet. [Internet] 4. Available from: http://www.ncbi.nlm.nih.gov/pmc/articles/PMC2265484/

Gagneux P, Boesch C, Woodruff DS. 1997. Microsatellite scoring errors associated with noninvasive genotyping based on nuclear DNA amplified from shed hair. Mol. Ecol. 6:861–868.

Gilbert MTP, Bandelt H-J, Hofreiter M, Barnes I. 2005. Assessing ancient DNA studies. Trends Ecol. Evol. 20:541–544.

Pöllath N, Peters J. 2011. "Smoke on the Mountain" - Animal Sacrifices for the Lord of Doliche. In: Winter E, editor. Von Kummuh nach Telouch. Asia Minor Studien. 64. Bonn: Habelt. p. 47–68.

Rubin C-J, Zody MC, Eriksson J, Meadows JRS, Sherwood E, Webster MT, Jiang L, Ingman M, Sharpe T, Ka S, et al. 2010. Whole-genome resequencing reveals loci under selection during chicken domestication. Nature 464:587–591.

Svensson EM, Anderung C, Baubliene J, Persson P, Malmström H, Smith C, Vretemark M, Daugnora L, Götherström A. 2007. Tracing genetic change over time using nuclear SNPs in ancient and modern cattle. Anim. Genet. 38:378–383.
